# Integrating Interprofessional Trainees into a Complex Care Program for Veterans Experiencing Homelessness: Effects on Health Services Utilization

**DOI:** 10.1007/s11606-021-06856-9

**Published:** 2021-09-30

**Authors:** Lillian Gelberg, Samuel T. Edwards, Elizabeth R. Hooker, Meike Niederhausen, Andrew Shaner, Brianna J. Cowan, Carole M. Warde

**Affiliations:** 1 Department of Family Medicine, David Geffen School of Medicine at UCLA, Los Angeles, CA USA; 2 Department of Health Policy and Management, UCLA Fielding School of Public Health, Los Angeles, CA USA; 3 Office of Healthcare Transformation and Innovation, VA Greater Los Angeles Healthcare System, Los Angeles, CA USA; 4 Center to Improve Veteran Involvement in Care, VA Portland Health Care System, Portland, OR USA; 5 Oregon Health & Science University (OHSU), Portland, OR USA; 6 Portland State University School of Public Health, Portland, OR USA; 7 VA Greater Los Angeles Healthcare System, Los Angeles, CA USA; 8 Department of Psychiatry, David Geffen School of Medicine at UCLA, Los Angeles, CA USA; 9 Department of Medicine, David Geffen School of Medicine at UCLA, Los Angeles, CA USA

**Keywords:** interprofessional, homeless, Veteran, ambulatory care, hospitalization

## Abstract

**Abstract:**

**PURPOSE:**

High-quality, comprehensive care of vulnerable populations requires interprofessional ambulatory care teams skilled in addressing complex social, medical, and psychological needs. Training health professionals in interprofessional settings is crucial for building a competent future workforce. The impacts on care utilization of adding continuity trainees to ambulatory teams serving vulnerable populations have not been described. We aim to understand how the addition of interprofessional trainees to an ambulatory clinic caring for Veterans experiencing homelessness impacts medical and mental health services utilization.

**METHODS:**

Trainees from five professions were incorporated into an interprofessional ambulatory clinic for Veterans experiencing homelessness starting in July 2016. We performed clinic-level interrupted time series (ITS) analyses of pre- and post-intervention utilization measures among patients enrolled in this training continuity clinic, compared to three similar VA homeless clinics without training programs from October 2015 to September 2018.

**RESULTS:**

Our sample consisted of 37,671 patient- months. There was no significant difference between the intervention and comparison groups’ post-intervention slopes for numbers of primary care visits (difference in slopes =−0.16 visits/100 patients/month; 95% CI −0.40, 0.08; *p*=0.19), emergency department visits (difference in slopes = 0.08 visits/100 patients/month; 95% CI −0.16, 0.32; *p*=0.50), mental health visits (difference in slopes = −1.37 visits/month; 95% CI −2.95, 0.20; *p*= 0.09), and psychiatric hospitalizations (−0.005 admissions/100 patients/month; 95% CI −0.02, 0.01; *p*= 0.62). We found a clinically insignificant change in medical hospitalizations.

**CONCLUSIONS:**

Adding continuity trainees from five health professions to an interprofessional ambulatory clinic caring for Veterans experiencing homelessness did not adversely impact inpatient and outpatient care utilization. An organized team-based care approach is beneficial for vulnerable patients and provides a meaningful educational experience for interprofessional trainees by building health professionals’ capabilities to care for vulnerable populations.

**Supplementary Information:**

The online version contains supplementary material available at 10.1007/s11606-021-06856-9.

## INTRODUCTION

In 2019, approximately 568,000 people experienced homelessness (PEH) in the USA on any given night, and that number continues to increase.^1^ PEH have high rates of chronic medical conditions, mental illness, and substance use disorders contributing to higher mortality than housed persons. ^2^ Historically poor access to primary care led to higher use of emergency and hospital care. Since 2009, the Veteran Health Administration’s national homeless program has reduced Veteran homelessness by nearly 50% using a multidisciplinary ambulatory care team approach with Housing First principles. Intensive ambulatory care teams composed of professionals from mental health, social services, and medicine successfully meet the complex needs of PEH.^3^ O’Toole et al. found that patients enrolled in the VA homeless patient-aligned care team (HPACT), a population-tailored multidisciplinary primary care clinic, had lower costs of care compared with primary care not targeted to the needs of Veterans experiencing homelessness (VEH). HPACT patients had more social work and primary care visits, and fewer emergency department visits, outpatient psychiatry visits, and hospitalizations.^4^

To optimally prepare future health professionals to care for vulnerable patients, trainees could be integrated into intensive ambulatory care teams targeting these patient populations,^5^ thereby improving access to care while also building the workforce’s skills and capacity to care for complex patients. A study of 5 VA sites showed that adding trainees to interprofessional (IP) primary care teams led to improved quality of diabetic care, compared to teams without trainees. ^6^ We do not know the impact of trainees on health care utilization by vulnerable PEH.

This study aims to determine if the integration of IP trainees into an ambulatory clinic for VEH impacts medical and mental health care services utilization. We integrated trainees from five professions into an existing VA HPACT, creating the first IP academic HPACT (IA-HPACT). We compared rates of outpatient, emergent, and inpatient visits by VEH of the IA- HPACT to similar HPACTs without a continuity IP training program. We hypothesized that health care utilization would not differ between patients of the IA-HPACT and patients of comparison HPACTs.

## METHODS

### Design

We performed clinic-level interrupted time series (ITS) analyses of outcomes among patients enrolled at the IA-HPACT before and after the start of the IP training program and compared them to outcomes at comparison HPACTs. The Veterans Health Administration provided a waiver of informed consent for this quality improvement activity exempt from institutional review board review.

### Setting

#### Interprofessional Academic Homeless Patient-Aligned Care Team (IA-HPACT)

The IA-HPACT clinic, based at a large urban VA medical center, serves approximately 3000 patients annually providing co-located social services and primary and mental health care. There are 2 large care teams each com- posed of 3–4 teamlets. Large care teams included a psychiatrist or psychiatric nurse practitioner, psychologist, pharmacist, social worker, and medical support assistant (clerk) who are shared by teamlets of a primary care provider (internal medicine physician or nurse practitioner), RN care manager, and LVN. Collaboration of IP care occurs during daily huddles, weekly case conferences, sequential visits with different providers, shared patient visits (both professions scheduled with patient simultaneously), warm hand-offs, and instant messaging.

Each year, new trainees from five professions establish continuity panels. Fifty-one trainees participated in the IP program during the first 3 years: 4 psychology fellows, 9 psychiatry residents, 4 pharmacy residents, 20 internal medicine (IM) residents, and 14 nurse practitioners (10 NP residents and 4 NP students). Trainees practiced in IA-HPACT for 1 year except for IM residents, who cared for continuity panels over 3 years. The IP trainees were integrated into the workflow of the whole clinic including primary care, mental healthcare, and pharmacy visits, and same-day visits for primary care and mental health.

We developed, implemented, and evaluated a competency- based IP curriculum relevant to all of the health professions trainees in IA-HPACT. The IP curriculum emphasized team- based care, humanism, well-being, relationship-centered communication, quality improvement, panel management, and social determinants of health as described in a separate paper. ^7^ We found the IA-HPACT curriculum led to improved trainee perspectives of team performance, team relationship skills, and effectiveness of meeting practices, while also avoiding burnout.

#### Comparison HPACT Sites

In the USA in 2018, 61 HPACTS served 19,000 patients. Four HPACTs were selected as comparison sites because they were most similar in (1) being a top-performing HPACT (based on primary care access, staffing, # visits, and access to housing programs); (2) having a strong physician director (whose primary worksite is HPACT and fully implements the HPACT model of care); and (3) being under the VA’s primary care administration. Some comparison sites offered educational experiences (i.e., rotations) for medical students and residents, but they did not incorporate trainees into continuity patient panels. Clinics at comparison sites were composed of similar disciplines.

### Measures

We analyzed measures of health care utilization routinely collected by the national HPACT program office, derived from the VA Corporate Data Warehouse from October 2015 to September 2018. Measures included numbers of visits to primary care (seen by assigned HPACT provider), mental health, emergency department, and admissions to general hospital (medicine, neurology, and surgery) and psychiatry services. Covariates included clinic panel size, proportion of panel patients at high risk of hospitalization (Care Assessment Needs Score > 90, i.e., CAN Score^8^), and proportion of panel patients with age over 65 years. Mental health visits consisted of a combination of general mental health, geropsychiatry, mental health intensive case management, psychosocial rehabilitation, post-traumatic stress disorder, residential rehabilitation, substance abuse, and vocational rehabilitation visits. As team assignment data were not available across sites over time, data were analyzed at the clinic level and reported monthly.

### Statistical Analysis

HPACT utilization measures were collected for 36 months from October 2015 to September 2018, with IA-HPACT trainees starting in July 2016 at month 10.

We calculated descriptive statistics and produced line plots to visually explore trends in outcome measures and covariates by site over time. We excluded one comparison site due to 6 months of missing data and unexplained widely varying values. The potential covariates of panel size, percentage of female, percentage of age >65, and percentage of panel with CAN score >90 were highly correlated with one another (all *p*<0.01), suggesting collinearity. We chose to retain percent- age of age >65 and percentage of panel with CAN score >90 in models due to their clinical relevance. All outcomes were transformed to the number of visits or admissions per 100 patients at the site per month.

To calculate the predicted slopes for each group during pre- and post-intervention periods, we ran unadjusted and adjusted multiple ITS models; adjusted models included percentage of panel with age older than 65 years and percentage of panel with CAN score greater than 90. We conducted ITS analyses using Newey ordinary least squares regression, with a maxi- mum lag order of autocorrelation set to 12 months. We used Stata’s margins command to obtain predicted values of each outcome by clinic, at a specific time point, given the model coefficients and each clinic’s actual observed values for their covariates. For the comparison sites, site values were averaged to give the predicted values for the comparison group. Graphical depiction shows a line graph that connects these predicted values. Analyses were conducted in Stata 16 (StataCorp, College Station, TX, USA). We used a 2-sided *p*<0.05 as a significance threshold.

## RESULTS

Our sample included clinic-level data for 4 HPACT sites over 36 months, for a total of *n*=37,671 patient-months. All outcomes were defined as the number of visits or admissions per 100 patients at the site per month.

Prior to program initiation, the intervention IA-HPACT on average saw more unique patients monthly than the three combined comparison sites as noted in Table 1 (3252.0 vs. 1473.9). The IA-HPACT also had a slightly higher proportion of patients over age 65 years old (19.3% vs. 14.5%), and a slightly lower proportion of patients with a CAN score of > 90 (27.4% vs. 32.1%).

**Table 1:** Average Monthly Characteristics of HPACT Patient Panels Prior to the IA-HPACT Training Program Implementation: IA-HPACT vs. Comparison Sites

Measure	IA-HPACT	Comparison sites (three sites combined)
Panel size	3252.0 (±103.1)	1473.9 (±26.6)
% patients with CAN > 90	27.4 (±1.3)	32.1 (±8.2)
% patients > 65 years old	19.3 (±1.0)	14.5 (±1.9)
% female	1.5 (±0.2)	3.1 (±1.6)

Data were collected monthly at the clinic level. The above figures represent the average of the months October 2015–June 2016, prior to the IA-HPACT starting (+/- standard deviation)

**Figure 1:**
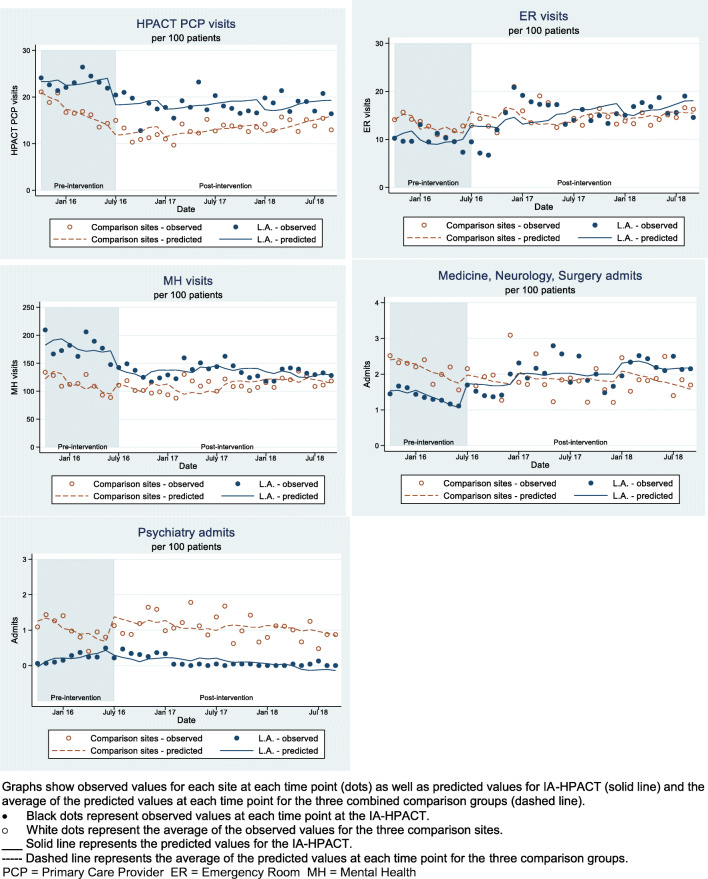
Health services utilization outcomes at the IA-HPACT and comparison HPACT clinics, from October 2015 to September 2018 (implementation of IA-HPACT training program in July 2016)

Figure 1 shows five outcomes at the IA-HPACT and comparison HPACTs before and after IA-HPACT program implementation, including observed values for each site at each time point, predicted values for IA-HPACT, and the average of the predicted values at each time point for the three combined comparison groups.

Post-intervention slopes are presented in Table 2. There was no significant difference between the intervention and comparison groups’ post-intervention slopes for primary care visits, emergency department visits, mental health visits, and psychiatric hospitalizations, indicating no detectable effect of IA-HPACT implementation on these outcomes. Specifically, primary care visits increased on average by 0.13 visits per 100 patients per month in IA-HPACT (95% CI −0.01, 0.27) and increased by 0.29 visits per 100 patients per month in comparison sites (95% CI 0.03, 0.54) with a difference in slopes of −0.16 visits/100 patients/month (95% CI −0.40; 0.08; *p*=0.19). For emergency department visits, the difference in slopes did not meet our significance threshold (0.08 visits/100 patients/ month −0.16, 0.32; *p*=0.50). The number of mental health visits in IA-HPACT decreased on average by 1.70 visits per 100 patients per month in the post-intervention period, while the comparison sites showed a non-significant decrease of 0.32 visits per 100 patients per month (difference in slopes −1.37; 95% CI −2.95; 0.20; *p*= 0.09). For general hospitalizations, there was a decrease at IA-HPACT of 0.004 hospitalizations per 100 patients per month (95% CI −0.02, 0.02), while at comparison HPACT sites, hospitalizations decreased by 0.04 hospitalizations per 100 patients per month (95% CI −0.06, −0.01). The difference in slopes was 0.03 (95% CI 0.01, 0.06; *p*=0.01). The post-intervention difference in slopes was close to null for psychiatry admissions (−0.005 admissions/100 patients/month; 95% CI −0.02, 0.01; *p*= 0.62).

**Table 2:** Change in Health Services Utilization Outcomes in HPACT Clinic Patients After IA-HPACT Training Program Implementation: IA-HPACT vs. Comparison Sites

	IA-HPACT	Comparison	Difference	*p*
HPACT primary care visits	0.13 (−0.01, 0.27)	0.29 (0.03, 0.54)	−0.16 (−0.40, 0.08)	0.19
Emergency department visits	0.15 (−0.08, 0.38)	0.07 (−0.14, 0.28)	0.08 (−0.16, 0.32)	0.50
Mental health visits	−1.70 (−2.77, −0.62)	−0.32 (−2.00, 1.35)	−1.37 (−2.95, 0.20)	0.09
Hospitalizations	−0.004 (−0.02, 0.02)	−0.04 (−0.06, −0.01)	0.03 (0.01, 0.06)	0.01
Psychiatry hospitalizations	−0.03 (−0.04, −0.02)	−0.03 (−0.04, −0.01)	−0.005 (−0.02, 0.01)	0.62

Measures expressed as slopes, i.e., the change in outcome per 100 patients/month (95% Confidence Interval).

Pre-intervention slopes and pre- and post-intervention means are presented in Appendix Table 1. Pre-intervention slopes were similar for ED visits, mental health visits, and general hospitalizations, but were different for primary care visits. Trends appeared similar by visual inspection (Appendix Figure 1).

## DISCUSSION

We assessed health services utilization among patients of an IP academic HPACT compared to non-academic HPACT sites with an interrupted time series analysis. We noted no significant difference in rates of primary care visits between the IA-HPACT and comparison sites. We also observed a trend toward lower rates of mental health visits in the IA-HPACT that did not meet statistical significance. We found no difference in rates of emergency department use and psychiatric hospitalization. We found that hospitalization rates decreased in both groups, but decreased more in the comparison HPACT settings. In summary, implementation of the IA-HPACT training program did not adversely change access to care, as measured by outpatient, emergent, and inpatient health services utilization.

This paper is the first to report the impact of trainees on health care utilization for PEH. Integrating inexperienced trainees into teams serving complex patients could elicit concern for adversely impacting patient continuity, access, and quality of care. Reassuringly, our findings show that adding trainees from multiple professions to IP complex care teams did not impact patients’ ability to access outpatient care or increase costly emergent and inpatient care. We attribute our findings to a high level of IP team care fostered by co- location of team disciplines, frequent meetings and unstructured communication for care coordination, the presence of clinical pharmacy, and team members’ willingness to proactively address patient care needs.

Our findings contribute to the limited literature on the impact of IP training programs on patient health care utilization.^6, 9^ The few studies assessing the effect on patient care outcomes of general ambulatory training programs have shown mixed findings. Patient satisfaction was not adversely affected and diabetes control benefited from management by trainees.^10^ Similarly, diabetes control of patients managed by interprofessional teams of trainees improved at 5 VA primary care sites. ^6^ In a walk-in clinic, patients of trainees and supervising attendings had similar patient satisfaction, symptom resolution, and functional status improvement.^11^ In contrast, one study showed that trainees deliver poorer quality of preventive care compared to faculty. ^12^

In general, the discontinuous schedules of trainees and faculty in academic ambulatory clinics^13, 14^ can make continuity of care and same-day clinic access challenging. We believe that our structured team-based approach, supported by organizational funding, workload management,^14^ and dedicated supervising faculty from multiple professions^7^, contributed to our findings.

### Limitations

This study analyzes aggregated clinic-level data and therefore has less power to detect site differences than individual patient-level analyses. However, most of the analyses did not reveal worse health services utilization outcomes at the IA-HPACT. IP curricula, dedicated faculty supervisors, and team organization at the IA-HPACT site may have enhanced the value of trainees locally. Therefore, our findings may not generalize to programs without the same level of organizational support or IP composition. Further, as an observational study, the selection of intervention and comparison sites was non-random. However, we selected comparison sites to be as similar as possible to intervention sites in team composition and patient population, and we used an interrupted time series design. Finally, these findings are specific to the VA’s primary care team model and may not apply to other health care systems.

## CONCLUSIONS

Our findings suggest that trainees care for the complex needs of vulnerable VEH without negatively impacting use of ambulatory, emergency, and hospital services. To increase future workforce capabilities, we propose that other VA and non-VA homeless clinics consider integrating health professions trainees longitudinally into their care teams. Future research should be conducted to replicate our findings regarding the impact of interprofessional ambulatory training programs for PEH within the VA HPACTs and the Health Care for the Homeless Program clinics in the USA.^15^

## Supplementary Information

ESM 1 (DOCX 233 kb)
