# Supplementary material for: Integrating Interprofessional Trainees into a Complex Care Program for Veterans Experiencing Homelessness: Effects on Health Services Utilization
Source: J Gen Intern Med. 2021 Sep 30;36(12):3659–64. doi: 10.1007/s11606-021-06856-9 (PMC8642561; doi:10.1007/s11606-021-06856-9)
Supplement: Supplementary file 1 — (DOCX 233 kb) [file 11606_2021_6856_MOESM1_ESM.docx]

**Online Appendix**

**Methods.** Mental Health Visit Definitions^a^

**Geropsych Visits:** Outpatient visits with stop codes* of 576, 577 or 578.

**Mental Health Visits:** Outpatient visits with stop codes of 592, 502, 505, 506, 509, 510, 512, 520, 521, 524, 525, 532, 533, 534, 538, 550, 553, 554, 557, 558, 559, 563, 564, 565, 566, 589 or 591.

**Mental Health Intensive Case Management Visits:** Outpatient visits with stop codes of 552 or 567.

**Psychosocial Rehab Visits:** Outpatient visits with stop codes of 582 or 583.

**Post Traumatic Stress Syndrome Visits:** Outpatient visits with stop codes of 516, 519, 540, 561, 562, 580 or 581.

**Residential Rehab Visits:** Outpatient visits with stop codes of 586, 587, 503, 593, 594, 595, 596, 725, 726, 727, 728, 729, 730, 731, 539, 588, 598 or 599.

**Substance Abuse Visits:** Outpatient visits with stop codes of 513, 514, 523, 547, 548 or 560.

**Vocational Rehab Visits:** Outpatient visits with stop codes of 535, 571, 572, 573 or 575.

**Stop codes are a VA specific identifier of different outpatient encounter types.*

*^a^ Department of Veterans Affairs. Homeless Patient Aligned Care Team Summary Report. Accessed 7/21/2017.*

**Results.** Detailed results by outcome: pre and post intervention predicted slopes and means with 95% confidence intervals, plots including observed vs. predicted values. Values expressed as per 100 patients/month.

**Outcome 1: HPACT primary care visits**

|  |  |
| --- | --- |

| Pre-intervention means | |  | Post-intervention means | |
| --- | --- | --- | --- | --- |
| IA-HPACT | 23.24 (22.75, 23.73) |  | IA-HPACT | 18.42 (17.98, 18.87) |
| Comparison sites | 17.22 (14.31, 20.14) |  | Comparison sites | 13.15 (11.01, 15.30) |
|  |  |  |  |  |
| Pre-intervention slopes | |  | Post-intervention slopes | |
| IA-HPACT | 0.22 (-0.13, 0.57) |  | IA-HPACT | 0.13 (-0.01, 0.27) |
| Comparison sites | -0.77 (-1.36, -0.17) |  | Comparison sites | 0.29 (0.03, 0.54) |
|  |  |  |  |  |
|  |  |  | **Post-intervention difference in slopes** | |
|  |  |  | **-0.16 (-0.40, 0.08)** | **p= 0.19** |

**Outcome 2: Emergency department visits**

|  |  |
| --- | --- |

| Pre-intervention means | |  | Post-intervention means | |
| --- | --- | --- | --- | --- |
| IA-HPACT | 10.04 (9.49, 10.59) |  | IA-HPACT | 15.20 (14.12, 16.29) |
| Comparison sites | 12.94 (8.09, 17.78) |  | Comparison sites | 14.77 (13.43, 16.10) |
|  |  |  |  |  |
| Pre-intervention slopes | |  | Post-intervention slopes | |
| IA-HPACT | 0.23 (-0.15, 0.61) |  | IA-HPACT | 0.15 (-0.08, 0.38) |
| Comparison sites | -0.31 (-1.38, 0.77) |  | Comparison sites | 0.07 (-0.14, 0.28) |
|  |  |  |  |  |
|  |  |  | **Post-intervention difference in slopes** | |
|  |  |  | **0.08 (-0.16, 0.32)** | **p= 0.50** |

**Outcome 3: Mental Health visits**

|  |  |
| --- | --- |

| Pre-intervention means | |  | Post-intervention means | |
| --- | --- | --- | --- | --- |
| IA-HPACT | 179.24 (173.94, 184.53) |  | IA-HPACT | 135.42 (133.18, 137.67) |
| Comparison sites | 113.06 (92.45, 133.68) |  | Comparison sites | 111.13 (100.04, 122.23) |
|  |  |  |  |  |
| Pre-intervention slopes | |  | Post-intervention slopes | |
| IA-HPACT | 0.43 (-2.54, 3.40) |  | IA-HPACT | -1.70 (-2.77, -0.62) |
| Comparison sites | -3.85 (-8.50, 0.80) |  | Comparison sites | -0.32 (-2.00, 1.35) |
|  |  |  |  |  |
|  |  |  | **Post-intervention difference in slopes** | |
|  |  |  | **-1.37 (-2.95, 0.20)** | **p= 0.09** |

**Outcome 4: Medicine Neurology or Surgery Hospital Admissions**

|  |  |
| --- | --- |

| Pre-intervention means | |  | Post-intervention means | |
| --- | --- | --- | --- | --- |
| IA-HPACT | 1.37 (1.35, 1.39) |  | IA-HPACT | 2.01 (1.91, 2.11) |
| Comparison sites | 2.13 (1.82, 2.45) |  | Comparison sites | 1.86 (1.73, 1.99) |
|  |  |  |  |  |
| Pre-intervention slopes | |  | Post-intervention slopes | |
| IA-HPACT | -0.07 (-0.10, -0.04) |  | IA-HPACT | -0.004 (-0.02, 0.02) |
| Comparison sites | -0.10 (-0.22, 0.02) |  | Comparison sites | -0.04 (-0.06, -0.01) |
|  |  |  |  |  |
|  |  |  | **Post-intervention difference in slopes** | |
|  |  |  | **0.03 (0.01, 0.06)** | **p= 0.01** |

**Outcome 5: Psychiatry Admits**

|  |  |
| --- | --- |

| Pre-intervention means | |  | Post-intervention means | |
| --- | --- | --- | --- | --- |
| IA-HPACT | 0.22 (0.20, 0.24) |  | IA-HPACT | 0.10 (0.02, 0.19) |
| Comparison sites | 1.01 (0.81, 1.22) |  | Comparison sites | 1.11 (0.99, 1.23) |
|  |  |  |  |  |
| Pre-intervention slopes | |  | Post-intervention slopes | |
| IA-HPACT | 0.07 (0.05, 0.09) |  | IA-HPACT | -0.03 (-0.04, -0.02) |
| Comparison sites | -0.08 (-0.15, -0.01) |  | Comparison sites | -0.03 (-0.04, -0.01) |
|  |  |  |  |  |
|  |  |  | **Post-intervention difference in slopes** | |
|  |  |  | **-0.005 (-0.02, 0.01)** | **p= 0.62** |
